# Supplementary material for: The interactome of intact mitochondria by cross-linking mass spectrometry provides evidence for coexisting respiratory supercomplexes
Source: Mol Cell Proteomics. 2017 Dec 8;17(2):216–32. doi: 10.1074/mcp.RA117.000470 (PMC5795388; doi:10.1074/mcp.RA117.000470)
Supplement: Supplemental Data [file supp_RA117.000470_133922_0_supp_23978_fzffwf.zip › spectra_annotation/mito_spectra_annotation/273-1-8-1-5-1.pdf]

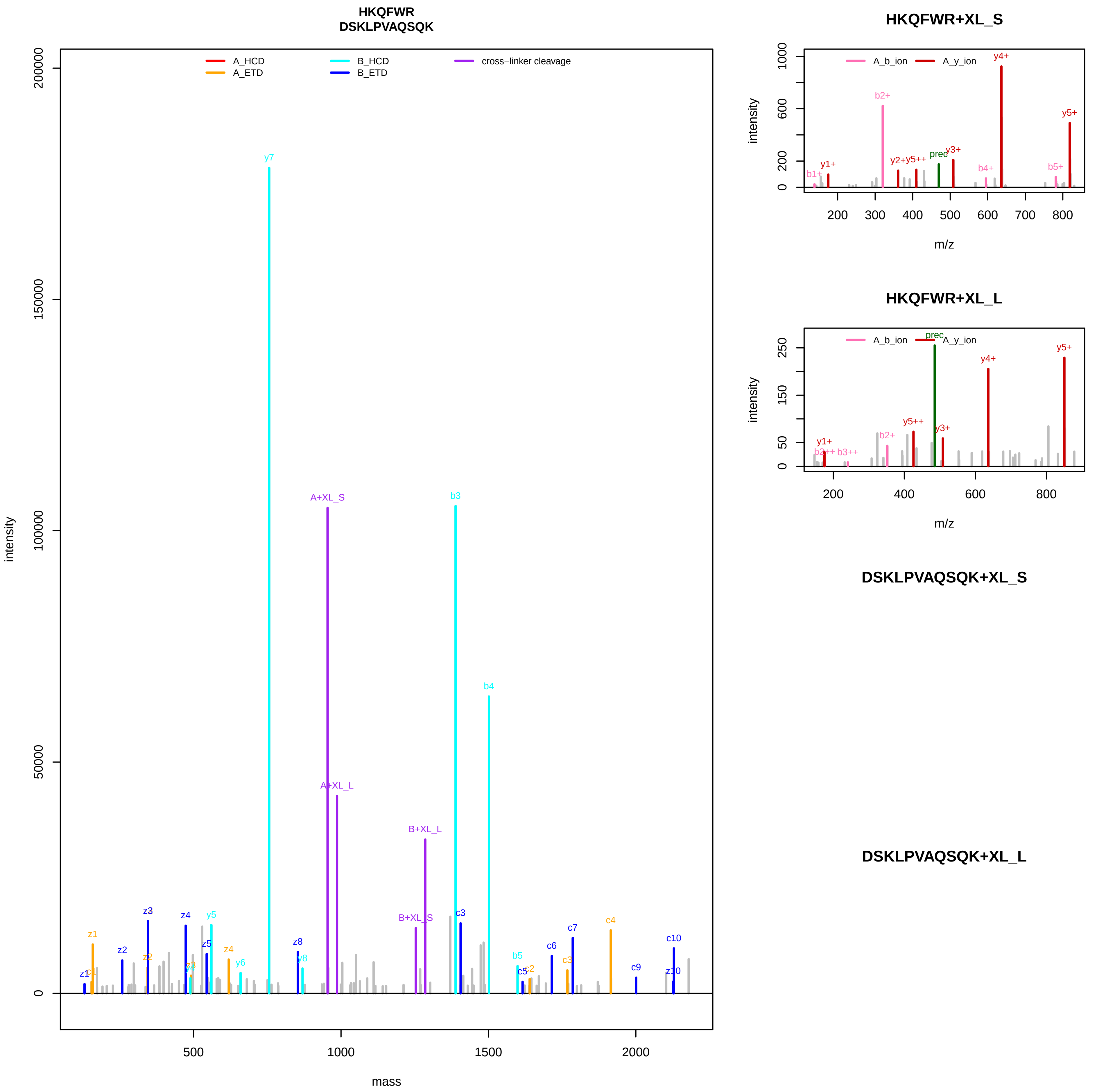
HKQFWR
DSKLPVAQSQK
A_HCD
A_ETD
B_HCD
B_ETD
cross−linker cleavage
intensity
mass
y7
A+XL_S
b3
b4
A+XL_L
B+XL_L
B+XL_S
c3
c4
c7
c6
c10
z3
z4
z1
z2
z8
z5
y5
y6
y8
z4
b5
c5
c2
c9
z10
HKQFWR+XL_S
A_b_ion
A_y_ion
y4+
b2+
y5+
y1+
b1+
y2+y5++
prec
y3+
b4+
b5+
m/z
HKQFWR+XL_L
y4+
y5+
y5++
y3+
b2+
y1+
b2++
b3++
DSKLPVAQSQK+XL_S
DSKLPVAQSQK+XL_L
